# Microstructural disruption of the right inferior fronto‐occipital and inferior longitudinal fasciculus contributes to WMH‐related cognitive impairment

**DOI:** 10.1111/cns.13283

**Published:** 2020-01-04

**Authors:** Hai‐Feng Chen, Li‐Li Huang, Hui‐Ya Li, Yi Qian, Dan Yang, Zhao Qing, Cai‐Mei Luo, Meng‐Chun Li, Bing Zhang, Yun Xu

**Affiliations:** ^1^ Department of Neurology Medical School and The State Key Laboratory of Pharmaceutical Biotechnology Institute of Brain Science Drum Tower Hospital Nanjing University Nanjing China; ^2^ Jiangsu Key Laboratory for Molecular Medicine Medical School of Nanjing University Nanjing China; ^3^ Jiangsu Province Stroke Center for Diagnosis and Therapy Nanjing China; ^4^ Nanjing Clinic Medical Center for Neurology Nanjing China; ^5^ Department of Radiology Afliated Drum Tower Hospital of Nanjing University Medical School Nanjing China

**Keywords:** automated fiber quantification, cognitive impairment, the right inferior fronto‐occipital fasciculus, the right inferior longitudinal fasciculus, white matter hyperintensity

## Abstract

**Aims:**

White matter hyperintensity (WMH) is the most common neuroimaging manifestation of cerebral small vessel disease and is related to cognitive dysfunction or dementia. This study aimed to investigate the mechanism and effective indicators to predict WMH‐related cognitive impairment.

**Methods:**

We recruited 22 healthy controls (HC), 25 cases of WMH with normal cognition (WMH‐NC), and 23 cases of WMH with mild cognitive impairment (WMH‐MCI). All individuals underwent diffusion tensor imaging (DTI) and a standardized neuropsychological assessment. Automated Fiber Quantification was used to extract altered DTI metrics between groups, and partial correlation was performed to assess the associations between WM integrity and cognitive performance. Furthermore, machine learning analyses were performed to determine underlying imaging markers of WMH‐related cognitive impairment.

**Results:**

Our study found that mean diffusivity (MD) values of several fiber bundles including the bilateral anterior thalamic radiation (ATR), the left inferior fronto‐occipital fasciculus (IFOF), the right inferior longitudinal fasciculus (ILF), and the right superior longitudinal fasciculus (SLF) were negatively correlated with memory function, while that of the anterior component of the right IFOF and the posterior and intermediate component of the right ILF showed significant negative correlation with MMSE and episodic memory, respectively. Furthermore, machine learning analyses showed that the accuracy of recognizing WMH‐MCI patients from the WMH populations was up to 80.5% and the intermediate and posterior components of the right ILF and the anterior component of the right IFOF contribute the most.

**Conclusions:**

Changes in the properties of DTI may be the potential mechanism of WMH‐related MCI, especially the right IFOF and the right ILF, which may become imaging markers for predicting WMH‐related cognitive dysfunction.

## 1 INTRODUCTION

Cerebral small vessel disease (CSVD) is an age‐related clinical syndrome, manifesting as abnormal mood and gait, lacunar infarction, cognitive dysfunction, and Parkinson's disease. MRI manifestations include lacunar infarction, white matter hyperintensities (WMHs), perivascular spaces, microbleeds, and brain atrophy.^1^ White matter hyperintensities is perceived as the most common neuroimaging manifestation because WMH is visible in 80% of healthy people over 60 years and almost all the people over 90 years.^2^ Different degrees of demyelination, gliosis and loss of fibers and oligodendrocytes are shown in the pathological examination of WMH.^3^, ^4^ Recent studies have found that WMH can lead to vascular cognitive impairment, and in some, may ultimately progress to dementia.^5^ The underlying mechanism is highly controversial. Vascular risk factor load, damage to neurotransmitter systems, interruption of prefrontal subcortical loops, and cerebral hypoperfusion were proposed to explain the correlation between WMHs and cognitive decline.^5^, ^6^, ^7^, ^8^, ^9^, ^10^, ^11^ However, patients with similar visual extensive WMH always manifested a variable severity of cognitive dysfunction and affected different cognitive domains.^12^ Longitudinal follow‐up studies also showed further deterioration of cognitive function over time in WMH patients with mild cognitive impairment.^13^ Furthermore, loss of microstructural integrity of normal‐appearing white matter (NAWM) was associated with executive function,^14^ which further suggested that WMH might be an extreme case of continuous spectrum of white matter (WM) damage. White matter tract disruption in diffusion tensor imaging (DTI) may lead to disconnections among cortico‐cortical or cortico‐subcortical pathways vital for some cognitive function. The so‐called “disconnection hypothesis” may play a role in WMH‐related cognitive impairment.^15^, ^16^

Diffusion magnetic resonance imaging (dMRI) is appropriate for WM microstructure study.^17^ Diffusion tensor imaging (DTI) is currently the only noninvasive method that can effectively observe and track the WM fiber tracts in a living human brain.^18^, ^19^ Fractional anisotropy (FA) and mean diffusivity (MD) are two quantitative measures of DTI that, respectively, detect the anisotropy and overall displacement of water molecule diffusion.^20^, ^21^ One approach to analyze DTI is voxel‐based analysis (VBA), the core of which is making measurements of FA or other diffusion metrics in specific regions of interest (ROIs), thus conducting group comparisons. For example, Della Nave reported that a large cluster of increased MD in the corpus callosum and pericallosal WM was associated with impaired motor function.^22^ It is noticeable that VBA is extremely sensitive to registration errors but may fail to achieve sufficient precision because of the diversity of tract sizes and shapes among individuals.^23^, ^24^ Tract‐based spatial statistics (TBSS) emerges then, which integrates selective voxels onto the nearest location on a pseudoanatomical WM skeleton and reduces the residual misalignment by 10% without spatial resolution loss.^25^ By using TBSS, Otsuka found that reduced diffusion anisotropy of the corpus callosum in patients with extensive leukoaraiosis may explain global cognitive impairment.^12^ However, it still fails to guarantee that any voxel corresponds to the same tract across individuals as mean FA equalizes the particularity by dispersing the original changes in specific sections of specific tracts to the whole bundle, with the average voxel value significantly influenced by the artificial split of anatomical locations.^25^, ^26^, ^27^ Consequently, what conventional MRI reveals is only the tip of the iceberg of the total SVD‐related WM damage and only when the disease has progressed to a serious extent can its neuroimaging markers be discovered. Tractography is thought to be the most accurate method for localizing fiber tracts at the individual level. Yet, there are still limitations: tractography depends on manual drawing of ROIs and averages diffusion properties on the entire WM tract.

Automated Fiber Quantification (AFQ), a new algorithm that automatically identifies WM tracts with defined waypoint ROIs and makes measurements at anatomically equivalent locations along their trajectories, has enabled correct delineation of the WM tracts and detailed information for analysis.^28^ Its superiority is mainly shown in four aspects: First, FA values vary at different locations within a tract but the shape of tract FA profile is still consistent among individuals; second, developmental alterations in FA can be localized to specific regions of fiber tracts; third, comparisons can be made between individual patients and healthy population norms to elucidate unique patient clinical features; finally, diverse behavioral outcomes can also be predicted by the behavioral tract profiles. Automated Fiber Quantification, serving as an alternative complementary method of VBA and TBSS, has been applied to provide new insights into WM degeneration in mild cognitive impairment in Alzheimer's disease patients.^29^ The effect of hypertension on WM integrity and the correlation between the destruction of structural architecture of WM and cognitive impairment have also been revealed by AFQ.

In view of its several advantages, we also consider AFQ as an ideal analytic method in our study. We hypothesized that DTI parameters vary among different positions on the same fiber bundle and the localization‐specific properties in WM integrity may contribute to WMH‐related cognitive impairment. Considering that vascular cognitive impairment is recognized as a progressive condition from normal cognitive status to frank dementia,^30^ early prediction and intervention play a pivotal role in the prevention of dementia, thus granting our study high significance.

## 2 MATERIALS AND METHODS

### 2.1 Participants

This is a cross‐sectional study approved by the Ethics Committee of Nanjing Drum Tower Hospital, and informed consent was obtained from all patients. Forty‐eight subjects with moderate‐to‐severe WMH and 22 healthy controls (HC) (age range 50‐80 years) were recruited from registration data of the Neurology Department in Drum Tower Hospital of Medical School, Nanjing University. Subjects with moderate‐to‐severe WMH were divided into WMH‐normal cognition (WMH‐NC, n = 25) and WMH‐mild cognitive impairment (WMH‐MCI, n = 23) based on neuropsychological assessment. Moderate‐to‐severe WMH was defined by neuroimaging evidence: WMH Fazekas scale 2 or 3.^31^, ^32^ Exclusion criteria included dementia (MiniMental State Examination [MMSE] <24), cerebral infarction, cerebral microbleeds, intracranial hemorrhage, nonvasculogenic WMH mimics (eg, multiple sclerosis), intra/extracranial large artery stenosis >50%, and other neurological or psychiatric disorders.

### 2.2 MRI scanning

All participants were examined on a Philips 3.0‐T scanner (Philips Medical Systems). The examination protocol included the high‐resolution T1‐weighted turbo gradient echo sequence (repetition time [TR] = 9.8 ms, flip angle [FA] = 8°, echo time [TE] = 4.6 ms, FOV = 250 × 250 mm^2^, number of slices = 192, acquisition matrix = 256 × 256, thickness = 1.0 mm), the FLAIR sequence (TR = 4.500 ms, TE = 333 ms, time interval [TI] = 1.600 ms, number of slices = 200, voxel size = 0.95 × 0.95 × 0.95 mm^3^, acquisition matrix = 270 × 260), and the diffusion‐weighted imaging sequence (TR = 9.154 ms, TE = 55 ms, acquisition matrix = 112 × 112, FOV = 224 × 224 mm^2^, thickness = 2.5 mm, voxel size = 2 × 2 × 2.5 mm^3^, the number of gradient directions = 32 (b = 1000 s/mm^2^) and one b0 image).

### 2.3 Neuropsychological measurement

All subjects underwent a standardized neuropsychological assessment protocol performed by an experienced neuropsychologist. General cognition was evaluated by the Beijing version of the Montreal Cognitive Assessment (MoCA‐BJ) and MMSE.^33^ As described in our previous study, MoCA‐BJ was used to detect WMH‐MCI according to the education level.^33^ In addition, a neuropsychological battery that included AVLT‐DR, Wechsler Memory Scale Visual Reproduction‐delayed recall (WMS‐VR‐DR), Category Verbal Fluency (CVF), Boston Naming Test (BNT), Trail Making Test‐A and Trail Making Test‐B (TMT‐A and TMT‐B) and Stroop Color and Word Tests A, B, and C (Stroop A, B, and C) was used to evaluate the multiple cognitive domains of episodic memory, language, executive function, and information processing speed. The raw examination scores were Z‐transformed to calculate each cognitive domain score.

### 2.4 Magnetic resonance image preprocessing

For diffusion images, the data preprocessing was carried out by FSL 5.0.9 software (Oxford Centre for Functional Magnetic Resonance Imaging of the Brain, University of Oxford; https://www.fmrib.ox.ac.uk/fsl/). The preprocessing included the following steps: DICOM‐to‐NIfTI format conversion, registering DWI images (b = 1000 s/mm^2^) to the non‐DWI image (B0), eddy current and head motion correction, and then nonbrain tissue exclusion. After preprocessing, whole‐brain images of diffusion metrics, including FA, MD, axial diffusivity (AD), and radial diffusivity (RD), were obtained via DTIFIT command of FSL.

### 2.5 Automated fiber quantification procedure

We identified whole‐brain WM fiber tracts (20 major fiber tracts) and further quantified the diffusion metrics along the tract trajectory by applying the AFQ package.^28^ A brief description of the steps required to obtain the AFQ results in this study is as follows: (a) The 3D T1‐weighted images were co‐registered into the b0 image for each participant based on FSL, and poorly aligned images were then excluded according to the visual assessment; (b) whole‐brain tractography using deterministic tractography with thresholds of turning angle < 30° and FA > 0.2; (c) waypoint regions of interest (ROI)‐based tract segmentation as described in the previous study; (d) fiber tract refinement based on the fiber tract probability maps; (e) fiber tract cleaning by a outlier rejection algorithm^28^; and (f) calculation of the diffusion measures (FA, MD, AD, and RD) along each fiber tract at 100 equidistant nodes. The identified 20 WM tracts in the whole brain are listed in Table 1. Because AFQ uses strict criterion for tract identification, it did not successfully identify all 20 WM tracts in each participant.^34^ We excluded 4 fiber tracts, the bilateral cingulum hippocampus (CH) and bilateral arcuate fasciculus (AF) which did not identify in large portion of subject (Table 1). Table 2 shows the detailed demographic and clinical information of participants in which AFQ successfully identified all 16 tracts, and only these participants (HC, n = 23; WMH‐NC, n = 19; WMH‐MCI, n = 22) are used for the further analyses.

**Table 1:** Successful identification rate for each of 20 fiber tracts in HC, WMH‐NC and WMH‐MCI

Index	Tract	Total subjects or raw samples (N0)				No. of subjects showing successful tract identification (N1)				Ratio (N1/N0)			
		HC	WMH‐NC	WMH‐MCI	Total	HC	WMH‐NC	WMH‐MCI	Total	HC (%)	WMH‐NC (%)	WMH‐MCI (%)	Total (%)
1	ATR_L	23	22	25	70	23	22	25	70	100	100	100	100
2	ATR_R	23	22	25	70	23	22	25	70	100	100	100	100
3	CST_L	23	22	25	70	23	22	25	70	100	100	100	100
4	CST_R	23	22	25	70	23	22	25	70	100	100	100	100
5	CC_L	23	22	25	70	23	21	25	69	100	95.5	100	98.6
6	CC_R	23	22	25	70	23	19	24	66	100	86.4	96.0	94.3
7	CH_L	23	22	25	70	19	13	20	52	82.6	59.1	80.0	74.3
8	CH_R	23	22	25	70	11	14	11	36	47.8	63.6	44.0	51.4
9	Forceps major	23	22	25	70	23	22	23	68	100	100	92.0	97.1
10	Forceps minor	23	22	25	70	23	22	25	70	100	100	100	100
11	IFOF_L	23	22	25	70	23	22	25	70	100	100	100	100
12	IFOF_R	23	22	25	70	23	22	25	70	100	100	100	100
13	ILF_L	23	22	25	70	23	22	25	70	100	100	100	100
14	ILF_R	23	22	25	70	23	22	25	70	100	100	100	100
15	SLF_L	23	22	25	70	23	22	25	70	100	100	100	100
16	SLF_R	23	22	25	70	23	22	25	70	100	100	100	100
17	UF_L	23	22	25	70	23	22	25	70	100	100	100	100
18	UF_R	23	22	25	70	23	22	25	70	100	100	100	100
19	AF_L	23	22	25	70	20	22	23	65	87.0	100	92.0	92.9
20	AF_R	23	22	25	70	19	17	19	55	82.6	77.3	76.0	78.6

Abbreviations: AF_L, left arcuate fasciculus; AF_R, right arcuate fasciculus; ATR_L, left anterior thalamic radiation; ATR_R, right left anterior thalamic radiation; CC_L, left cingulum cingulate; CC_R, right cingulum cingulate; CH_L, left cingulum hippocampus; CH_R, right cingulum hippocampus; CST_L, left corticospinal tract; CST_R, right corticospinal tract; HC, health control; IFOF_L, left inferior fronto‐occipital fasciculus; IFOF_R, right inferior fronto‐occipital fasciculus; ILF_L, left inferior longitudinal fasciculus; ILF_R, right inferior longitudinal fasciculus; MCI, mild cognitive impairment; NC, normal cognition; SLF_L, left superior longitudinal fasciculus; SLF_R, right superior longitudinal fasciculus; UF_L, left uncinate fasciculus; UF_R, right uncinate fasciculus; WMH, white matter hyperintensities.

**Table 2:** Demographic and neuropsychological data

Items	HC (n = 23)	WMH		*F*/*χ* ^2^	*P*	Post hoc analyses		
		NC (n = 19)	MCI (n = 22)			HC VS WMH‐NC	HC VS WMH‐MCI	WMH‐NC VS WMH‐MCI
Demographics								
Age (y)	62.3 ± 1.50	67.1 ± 1.66	65.1 ± 1.54	2.311	.108^b^	–	–	–
Education (y)	12.7 ± 0.67	11.7 ± 0.73	11.00 ± 0.68	1.599	.210^b^	–	–	–
Gender (male/female)	12/11	9/10	9/13	0.576	.750^a^	–	–	–
General cognition								
MMSE	29.2 ± 0.31	28.6 ± 0.34	27.3 ± 0.32	9.653	<.001^b^, *	0.206	<0.001*	0.006*
MoCA‐BJ	26.7 ± 0.36	26.4 ± 0.40	21.5 ± 0.37	61.959	<.001^b^, *	0.667	<0.001*	<0.001*
Composition Z scores of each cognitive domain								
Episodic Memory	0.45 ± 0.14	0.05 ± 0.16	−0.51 ± 0.15	11.137	<.001^b^, *	0.065*	<0.001*	0.011*
AVLT‐DR	0.35 ± 0.19	0.32 ± 0.21	−0.64 ± 0.19	8.626	.001^b^, *	0.896	<0.001*	0.001*
VR‐DR (WMS)	0.54 ± 0.19	−0.22 ± 0.21	−0.37 ± 0.20	6.192	.004^b^, *	0.011*	0.002*	0.593
Information Processing Speed	0.27 ± 0.17	0.18 ± 0.19	−0.44 ± 0.18	4.816	.011^b^, *	0.720	0.005*	0.020*
TMT‐A (inverse)	0.45 ± 0.20	−0.02 ± 0.22	−0.45 ± 0.2	5.138	.009^b^, *	0.120	0.002*	0.142
Stroop A (inverse)	0.20 ± 0.20	0.24 ± 0.22	−0.41 ± 0.21	3.026	.056^b^	–	–	–
Stroop B (inverse)	0.17 ± 0.20	0.31 ± 0.22	−0.45 ± 0.21	3.729	.030^b^, *	0.640	0.035*	0.015*
Language	0.25 ± 0.13	−0.08 ± 0.19	−0.19 ± 0.18	1.678	.195	–	–	–
CVF	0.01 ± 0.21	0.11 ± 0.23	−0.11 ± 0.22	0.229	.796	–	–	–
BNT	0.48 ± 0.20	−0.26 ± 0.22	−0.28 ± 0.20	4.661	.013^b^, *	0.014*	0.009*	0.961
Executive Function	0.20 ± 0.14	0.12 ± 0.16	−0.32 ± 0.14	3.741	.029^b^, *	0.581	<0.001*	<0.001*
DST‐backward	0.11 ± 0.21	0.02 ± 0.23	−0.13 ± 0.22	0.320	.727	–	–	–
TMT‐B (inverse)	0.52 ± 0.19	−0.01 ± 0.21	−0.54 ± 0.19	7.490	.001^b^, *	0.239	<0.001*	<0.001*
Stroop C (inverse)	−0.02 ± 0.21	0.35 ± 0.26	−0.28 ± 0.21	2.152	.125	–	–	–

Note

Values are presented as the mean ± standard error (SE).

Abbreviations: AVLT‐DR, Auditory Verbal Learning Test‐delayed recall; BNT, Boston Naming Test; CVF, category verbal fluency; DST, Digit Span Test; HC, health control; MCI, mild cognitive impairment; MMSE, mini mental state examination; MoCA‐BJ, Beijing version of the Montreal Cognitive Assessment; NC, normal cognition; Stroop A, B and C, Stroop Color and Word Tests A, B, and C; TMT‐A and TMT‐B, Trail Making Test‐A and B; VR‐DR, visual reproduction‐delay recall; WMH, white matter hyperintensities; WMS, Wechsler Memory Scale.

a The p value was obtained by *χ* ^2^ test.

b The p value was obtained by one‐way ANOVA.

* Indicates a statistical difference between groups, *P* < .05.

### 2.6 Statistical and machine learning analyses

Demographic characteristics (including age, years of education, and gender distribution) and cognitive assessment were compared using one‐way analysis of variance (ANOVA) or the chi‐squared (*χ*2) test in SPSS software (Version 22). Significance was set at *P* < .05.

To examine group difference of WM tracts, we calculated DTI metrics (FA, MD, AD, and RD) of each fiber tract by averaging diffusion values of 100 nodes along each WM tract and performed the ANOVA to determine the between‐group differences. Age, years of education, and gender were controlled for as confounding covariates.

Next, the point‐wise analyses were based on the “Randomize” program of FSL software. Age, years of education, and gender were included as covariates in the general linear model (GLM). Family‐wise error (FWE) correction was applied to the 1600 points (16 fibers × 100 points) and a corrected significant level at 0.05.^35^ Then, within each fiber, only more than or equal to three adjacent nodes were reported.^36^ To assess the associations between WM integrity and cognitive performance, partial correlation was performed using SPSS while controlling for age, years of education, and gender.

Finally, we used a random forest (RF) classifier to identify the included neuroimaging characteristics that best classify the WMH‐MCI and WMH‐HC. In this study, only those features that were significantly different between groups (including the tract level and point‐wise level) were entered the analysis. In this study, RF was built with 500 trees in the forest.^37^ Each decision tree is constructed using a bootstrap sample from the training data. The out‐of‐bag (OOB) observations were then predicted to obtain the unbiased estimate of the classification error. The advantage of the fit of the RF was assessed by averaging the individual tree classification errors. Then, the RF framework estimated the importance of a variable by seeking how much the classification errors increased when the OOB data for that variable were permuted while all others were left unchanged. Furthermore, we ranked the variables' importance by assigning a score to each covariate based on the ability to classify correctly the dependent label (WMH‐NC vs. WMH‐MCI). The diagnostic performance of the best MRI features according to the RF classifier was evaluated based on accuracy, sensitivity, and specificity.

## 3 RESULTS

### 3.1 Demographic and clinical characteristics

Demographic and clinical characteristics for the HC and WMH subgroups (WMH‐NC and WMH‐MCI) are provided in Table 2. There was no significant difference for age, years of education, and gender distribution between the subgroups (*P* > .05). The WMH‐MCI subgroup showed poorer performances on MMSE (*P* < .001), MoCA‐BJ (*P* < .001), episodic memory (*P* < .001), information processing speed (*P* < .011), and executive function (*P* < .029) than the other subgroups (the detailed cognitive assessment is shown in Table 2).

### 3.2 Group difference in WM tract and point‐wise levels

Between‐group difference of WM tract and point‐wise alterations was determined by mean diffusion metrics (FA, MD, AD, and RD) with AFQ.

#### 3.2.1 FA

We found that mean FA values of WM tracts significantly changed between the three groups in the bilateral anterior thalamic radiation (ATR), forceps minor, bilateral IFOF, and left ILF (Table S1 and Figure S1). Further analysis indicated that WMH‐NC showed significantly decreased FA in the bilateral ATR and forceps minor compared to the HC group. For the bilateral ATR, forceps minor, bilateral IFOF and left ILF, WMH‐MCI displayed lower FA in comparison with HC. However, no significant group difference was found between WMH‐NC and WMH‐MCI. In the point‐wise level, there were no obvious alterations in FA between HC, WMH‐NC, and WMH‐MCI (FWE correction, *P* > .05).

#### 3.2.2 MD

Apart from the left cingulum cingulate (CC), forceps major, and left uncinate fasciculus (UF), the remaining 13 fiber tracts exhibited significant difference in MD between HC, WMH‐NC, and WMH‐MCI (Table S2 and Figure S2). Compared to HC, WMH‐NC had significantly increased MD in the bilateral ATR, right corticospinal tract (CST), right CC, forceps minor, bilateral IFOF, bilateral ILF, bilateral superior longitudinal fasciculus (SLF), and right UF. In addition, WMH‐MCI showed higher MD relative to HC in the bilateral ATR, bilateral CST, right CC, forceps minor, bilateral IFOF, bilateral ILF, bilateral SLF, and right UF. Furthermore, the mean MD in the right ATR, right IFOF, right ILF, and left SLF was significantly different between WMH‐NC and WMH‐MCI.

**Figure 1:**
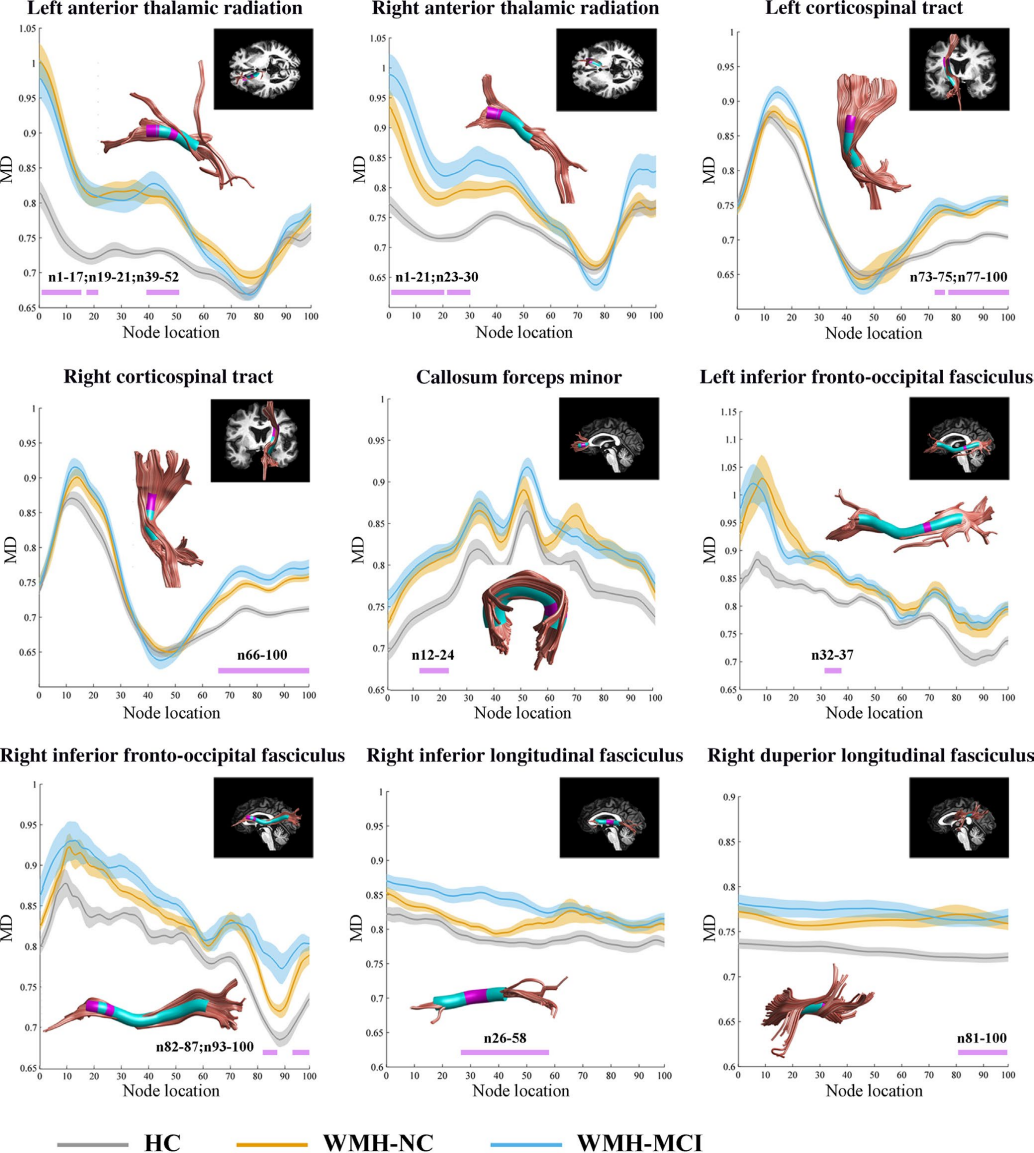
Significantly changed locations of fiber tracts in point‐wise comparison of MD profiles (FWE correction, *P* < .05). Abbreviation: HC, health control; NC, normal cognition; MCI, mild cognitive impairment; WMH, white matter hyperintensities; MD, mean diffusivity

In point‐wise comparison of MD profiles, significantly changed locations of fiber tracts (FWE correction, *P* < .05) were demonstrated as follows: (a) the anterior and intermediate component of the left ATR (nodes 1‐17; nodes 19‐21; nodes 39‐52); (b) the anterior component of the right ATR (nodes 1‐21; nodes 23‐30); (c) the superior portion of the left CST (nodes 73‐75; nodes 77‐100); (d) the superior portion of the right CST (nodes 66‐100); (e) the frontal lobe portion of the forceps minor (nodes 12‐24); (f) the posterior component of the left IFOF (nodes 32‐37); (g) the anterior component of the right IFOF (nodes 82‐87; nodes 93‐100); (h) the posterior and intermediate component of the right ILF (nodes 26‐58); and (i) the posterior component of the right SLF (nodes 81‐100) (Figure 1). Further post hoc multiple comparisons are listed in Table S3. It should be noted that WMH‐MCI obtained significantly higher mean MD values compared to WMH‐NC in the anterior component of the right ATR (nodes 1‐21), the superior portion of the right CST (nodes 66‐100), the anterior component of the right IFOF (nodes 82‐87; nodes 93‐100), and the posterior and intermediate component of the right ILF (nodes 26‐58).

#### 3.2.3 AD

Table S4 shows mean AD values of each WM fiber tract for each subject group. The bilateral ATR, bilateral CC, forceps minor, bilateral IFOF, right ILF, and right SLF exhibited significant differences between HC, WMH‐NC, and WMH‐MCI. Further statistical analyses are also listed in Table S4.

The point‐wise comparison of AD profiles between the three groups showed significant alterations (FWE correction, *P* < .05) mainly in the anterior and intermediate component of the left ATR (nodes 2‐14; nodes 43‐45), the anterior component of the right ATR (nodes 2‐15), the posterior component of the right CC (nodes 24‐30), and the anterior component of the right IFOF (nodes 84‐86) (Figure S3). Further multiple comparisons are listed in Table S3.

#### 3.2.4 RD

Table S5 shows that the three groups exhibited significant differences in RD in the bilateral ATR, right CST, forceps minor, bilateral IFOF, bilateral ILF, bilateral SLF, and right UF. Further multiple comparisons are also listed in Table S5.

In point‐wise comparison of RD profiles, significantly changed locations of fiber tracts (FWE correction, *P* < .05) were demonstrated as follows: (a) the anterior and intermediate component of the left ATR (nodes 1‐12; nodes 42‐51); (b) the anterior and intermediate component of the right ATR (nodes 1‐19; nodes 44‐49); (c) the inferior portion of the left CST (nodes 23‐26); (d) the superior portion of the right CST (nodes 93‐100); (e) the anterior component of the right IFOF (nodes 92‐94); and (f) the posterior and intermediate component of the right ILF (nodes 32‐50) (Figure S4). Further post hoc analyses are listed in Table S3.

### 3.3 Correlations between altered diffusion metrics and cognition

**Figure 2:**
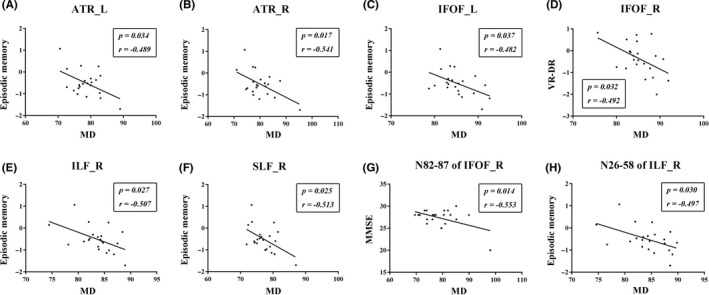
The correlations between MD values and cognition assessment in WMH‐MCI. A‐C and E‐F, Significantly negative correlations between episodic memory and the mean MD of the following tracts: left ATR (*r* = −.489, *P* = .034), right ATR (*r* = −.541, *P* = .017), left IFOF (*r* = −.482, *P* = .037), right ILF (*r* = −.507, *P* = .027), and right SLF (*r* = −.513, *P* = .025). D, The mean MD of the right IFOF correlated negatively with VR‐DR (*r* = −.492, *P* = .032). G, The MD values in the anterior component of the right IFOF (nodes 82‐87) were negatively associated with MMSE (*r* = −.553, *P* = .014). H: The right ILF showed significant negative correlation with episodic memory in the posterior and intermediate component (nodes 26‐58) (*r* = −.497, *P* = .030). MCI, mild cognitive impairment; WMH, white matter hyperintensities; MD, mean diffusivity; axial diffusivity; ATR_L, left anterior thalamic radiation; ATR_R, right anterior thalamic radiation; IFOF_L, left inferior fronto‐occipital fasciculus; IFOF_R, right inferior fronto‐occipital fasciculus; ILF_R, right inferior longitudinal fasciculus; SLF_R, right superior longitudinal fasciculus; MMSE, Mini Mental State Examination; VR‐DR, Visual Reproduction‐delayed recall

For the altered WM fiber tracts in Table S1, we found no significant correlation between FA and cognitive performance in WMH‐MCI. Next, we estimated the relationships between the other three diffusion metrics (MD, AD, and RD) and the cognition in WMH‐MCI. We summarized the correlations between MD values (the entire WM fiber tract in Table S2 and point‐wise level in Table S3 and cognition assessment in WMH‐MCI (Figure 2). We found significantly negative correlations between episodic memory and the mean MD of the following tracts: left ATR (*r* = −.489, *P* = .034), right ATR (*r* = −.541, *P* = .017), left IFOF (*r* = −.482, *P* = .037), right ILF (*r* = −.507, *P* = .027), and right SLF (*r* = −.513, *P* = .025). The mean MD of the right IFOF correlated negatively with VR‐DR (*r* = −.492, *P* = .032). In the point‐wise level, the MD values in the anterior component of the right IFOF (nodes 82‐87) were negatively associated with MMSE (*r* = −.553, *P* = .014). In addition, the right ILF showed significant negative correlation with episodic memory in the posterior and intermediate component (nodes 26‐58) (*r* = −.497, *P* = .030). AD and RD's correlations with cognition were also explored, as seen in Figure S5 and Figure S6.

### 3.4 Discrimination analysis

**Figure 3:**
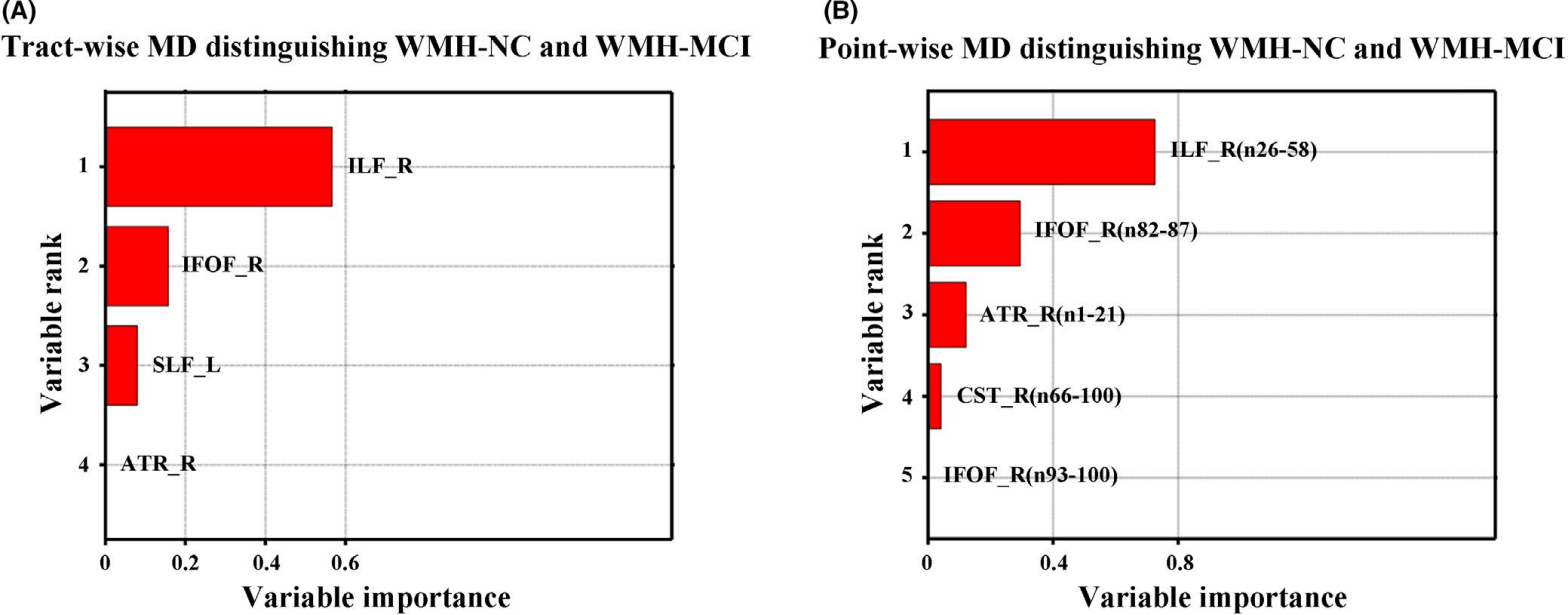
The variable importance differentiating WMH‐MCI from patients with WMH based on tract‐wise MD (A) and point‐wise MD (B). NC, normal cognition; MCI, mild cognitive impairment; WMH, white matter hyperintensities; MD, mean diffusivity; ATR_R, right anterior thalamic radiation; CST_R, right corticospinal tract; IFOF_R, right inferior fronto‐occipital fasciculus; ILF_R, right inferior longitudinal fasciculus; SLF_L, left superior longitudinal fasciculus

Table 3 shows the results of the RF analyses for identifying WMH‐MCI from patients with WMH based on diffusion measures in WM tract and point‐wise levels. Among these results, the point‐wise MD profile could be used to distinguish WMH‐MCI and WMH‐NC with the highest discrimination ability (accuracy = 80.5%; sensitivity = 81.8%; specificity = 79.0%). Furthermore, the importance in classifying diagnosis was achieved by MD measures of the ILF_R (n26‐58), followed by the IFOF_R (n82‐87), ATR_R (n1‐21), CST_R (n66‐100), and IFOF_R (n93‐100) (Figure 3B). The variable importance differentiating WMH‐MCI from patients with WMH based on other diffusion measures (MD [the tract level], AD, and RD) are presented in Figure 3A and Figure S7.

**Table 3:** Accuracy, sensitivity, and specificity of the discrimination analyses derived from the RF between WMH‐NC VS WMH‐MCI

Diffusion measures & fiber tracts	Accuracy (%)	Sensitivity (%)	Specificity (%)
FA			
–	–	–	–
MD			
ATR_R	68.3	77.3	57.9
IFOF_R			
ILF_R			
SLF_L			
ATR_R(n1‐21)	80.5	81.8	79.0
CST_R(n66‐100)			
IFOF_R(n82‐87)			
IFOF_R(n93‐100)			
ILF_R(n26‐58)			
AD			
ATR_R	65.9	68.2	63.2
CC_R			
IFOF_R			
ATR_R(n2‐15)	61.0	63.6	57.9
CC_R(n24‐30)			
IFOF_R(n84‐86)			
RD			
ATR_R	56.1	59.1	52.6
IFOF_R			
ILF_L			
ILF_R			
ATR_R(n1‐19)	73.2	77.3	68.4
IFOF_R(n92‐94)			
ILF_R(n32‐50)			

Abbreviations: AD, axial diffusivity; ATR_R, right anterior thalamic radiation; CC_R, right cingulum cingulate; CST_R, right corticospinal tract; FA, fractional anisotropy; IFOF_R, right inferior fronto‐occipital fasciculus; ILF_L, left inferior longitudinal fasciculus; ILF_R, right inferior longitudinal fasciculus; MCI, mild cognitive impairment; MD, mean diffusivity; NC, normal cognition; RD, radial diffusivity; RF, random forest; SLF_L, left superior longitudinal fasciculus; WMH, white matter hyperintensities.

## 4 DISCUSSION

Diffusion tensor imaging is the only noninvasive method to measure the microstructural integrity of brain tissue by detecting the diffusion of water molecules in it. Fractional anisotropy, MD, AD, and RD are four common indicators. White matter hyperintensities is the most common neuroimaging manifestation of CSVD, which is related to cognitive dysfunction or dementia. Studies that focused on NAWM have also confirmed that the change in DTI metrics of NAWM always precedes the development of WMH and is associated with MCI.^14^, ^38^ Combined with the emergence of WMH penumbra theory,^39^ we propose that direct visual WMH may be a manifestation of more extensive and subtle WM microstructural degeneration and those changes of the properties in DTI may be the potential mechanism of WMH‐related MCI, as stated in the theory of cortical “disconnection” hypothesis.^15^, ^16^ To further explore and verify the mechanism, many efforts have been made using VBA or TBSS.^12^, ^22^ However, their results are inconsistent and controversial and were obtained from a perspective of brain region or the entire fiber tract only. So we conducted the research, and in this study, we first found: (a) the abnormal WM bundles in WMH patients by using the AFQ algorithm; (b) the correlation between the WM injury and cognitive impairment; and (c) the candidate fiber tract as an imaging marker for identifying WMH‐related cognitive dysfunction.

First, at the overall level of fiber bundles, FA decreased in only 6 of 16 patients with WMH compared with normal subjects; yet the difference between the WMH‐NC and WMH‐MCI subgroups did not reach statistical significance. However, MD increased in 13 of 16 fiber bundles in the WMH groups in comparison with the NC. Furthermore, four bundles showed a statistically significant difference between the WMH‐MCI and WMH‐NC, that is, the right ATR, the right IFOF, the right ILF, and the left SLF. This suggests that MD is more sensitive than FA in detecting the degree of WM damage, consistent with the study on Alzheimer's disease.^40^, ^41^ The development of WMH is partly caused by focal ischemia, which may result in a decrease in tissue density and an increase in water diffusivity while maintaining underlying directional structure, and those outcomes cause an increase in MD when FA remains unchanged.^41^, ^42^

Partial correlation analysis showed that the MD values of several fiber bundles (left ATR, right ATR, left IFOF, right ILF, and right SLF) were negatively correlated with memory function in the WMH‐MCI group, which indicated that the more serious the damage with WM microstructure, the worse is the memory ability. Many efforts have been made to explore the correlation between WM microstructure and cognitive function, but some controversies remain. One reason is inconsistencies in detailed anatomical definitions like IFOF whose origin and termination of fiber tract have not been determined accurately.^43^ It was widely accepted that the IFOF connected the fronto‐marginal gyrus and lateral orbito‐frontal gyrus with the inferior occipital gyrus, the inferior part of the middle occipital gyrus, and lingual gyrus,^44^ but others proposed that the middle and posterior temporal lobes should be the posterior terminations of the IFOF.^45^ Regarding this, postmortem dissection and DTI techniques suggested that the bundle should be segmented into different subcomponents which can give us a more comprehensive understanding about its connectional architectures and their relevance to cognitive domains.^43^, ^46^ For example, the damage of IFOF‐1 originating from the polar part of the frontal lobe may explain its connection with memory function here. Besides, the natural crossing of the IFOF with other tracts like the ILF, SLF, and arcuate fascicle also makes the anatomy and function of certain bundles confusing.^43^ The FA values of the crossing regions of the posterior portion of the bilateral IFOs with the bilateral CSTs became higher when most of the affected regions from NC to MCI to AD had a decreased FA 40; these results also call for a more location‐specific approach for further research. Many studies obtained similar results suggesting that the integrity of ILF and SLF have an impact on memory.^47^, ^48^, ^49^ However, limited clinical data exist to inform the influence of ATR on episodic memory, while in rat experiments, it was found that rats with anterior thalamic lesions have difficulties with reference and working memory^50^; thus, more research is needed to verify its reliability.

Discrimination analysis was performed to identify WMH‐MCI from patients with WMH‐NC with MD values of the above four bundles; the accuracy was 68.29% and the right ILF and right IFOF contributed the most.

Next, we analyzed the FA and MD value of the 100 nodes segmented by the AFQ algorithm in each tract among the three groups. As a result, the FA did not vary from each other after FWE correction. Interestingly, nine fiber tracts have one or more specific regions where the MD clearly varied, with five regions showing a statistically significant difference between the WMH‐NC and the WMH‐MCI subgroups (the anterior component of the right ATR [nodes 1‐21], the superior portion of the right CST [nodes 66‐100], the anterior component of the right IFOF [nodes 82‐87; nodes 93‐100], and the intermediate and posterior component of the right ILF [nodes 26‐58]), indicating that the degree of WM microstructure injury is not constant along the whole tract, and some locations are more vulnerable than others, which resembles Zhang's finding that the abnormalities of nerve fiber tracts are localized to specific regions of the tracts in MCI and AD patients.^29^ The changes of some small "clumps" might have been covered up by analyzing the whole fiber bundle, which can explain the low accuracy of our results above.

Partial correlation analysis of cognitive function and diffusion parameters found that the MD values of the anterior component of the right IFOF (nodes 82‐87) were negatively associated with MMSE. One simple conjecture that the right IFOF became the first victim was due to its anatomical position. Though controversial, it is already known that the IFOF stretches a long distance as one of the major long‐range association tracts integrating the anatomically distal cortex, connecting occipito‐temporal (and parietal) areas to the frontal lobe through the external/extreme capsule region^51^, ^52^ which makes it more vulnerable to injury than other bundles. The polar part of the frontal lobe (BA10) is associated with many aspects of complex cognitive functions including episodic memory, social cognition, attention and multitasking and subcomponent analysis of IFOF gave the suggestion that these significant functions are primarily supported by WM connectivity between BA10 and IFOF.^43^, ^53^ Thomas's finding that only the reduction in tract integrity of the right ILF and the right IFOF can lead to the face recognition impairment suggested that the right hemisphere is more prominent in some cognitive domains.^54^

We also found that the right ILF showed a significant negative correlation with episodic memory in the posterior and intermediate component (nodes 26‐58). The ILF links the anterior temporal lobe with the extrastriate cortex of the occipital lobe, running along the lateral and inferior wall of the lateral ventricle and was found to be correlated with semantic autobiographical memory.^55^ Diffusion tensor imaging studies found that damaged microstructures of the ILF were significantly correlated with decreased memory function,^48^, ^55^ indicating that memory function depends on the connection integrity of the temporal lobe with other lobes such as the occipital lobe. What is interesting in Zemmoura's finding is that it is the posterior portion of the ILF instead of the anterior portion that plays a prominent role in reading impairment,^56^ which is consistent with our result that the posterior component of ILF was more vulnerable to injury. It also gave us a hint that different locations of the same fiber may be responsible for different cognitive domains. Despite these hypotheses, more work needs to be done to confirm the actual mechanism behind the phenomenon.

By using random forest classifier, the accuracy to recognize WMH‐MCI patients from the WMH populations was up to 80.5% at the level of node with the intermediate and posterior component of the right ILF (nodes 26‐58) and the anterior component of the right IFOF (nodes 82‐87) having the most identification effect. The vulnerable characteristics of ILF and IFOF in WMH are supported by Seiler who found that five tracts including forceps major, posterior thalamic radiation, inferior fronto‐occipital fasciculus, forceps minor, and inferior longitudinal fasciculus were particularly prone to WMH occurrence.^57^ Besides, many studies have confirmed the relationship between the integrity of ILF/IFOF and multiple cognitive functions including memory, reading, attention, and visual processing.^43^, ^48^, ^58^, ^59^ In conclusion, both the level of fiber bundles and the node level suggest that ILF and IFOF are key WM fiber tracts mediating certain cognitive functions and can be used as imaging markers for early recognition of WMH‐related cognitive impairment.

In addition to FA and MD, other diffusion metrics like AD and RD were also employed in our study. AD reflects diffusion parallel to axonal fibers which may reflect axonal injury and RD captures perpendicular diffusion which is linked to demyelination.^42^ AD values of ATR_L, ATR_R, and ILF_R were inversely correlated with episodic memory, while that of IFOF_R and ILF_R had a negative correlation with MoCA and executive function, respectively. Less correlation existed between RD values and cognitive function, only the RD values of ATR_R and SLF_R were negatively correlated with memory function. The accuracy of distinguishing WMH patients with cognitive dysfunction by AD values and RD values of altered fiber segments is lower than that of MD values, which are 61.0% and 73.2%, respectively. AD and RD are two parameters somewhat related to MD in that they are all derived from the three identical eigenvalues of the tensor (λ1, λ2, and λ3),^60^ higher sensibility of MD on catching WM degeneration had been found in Alzheimer's disease.^40^, ^42^ We came to the same conclusion and also found that their ability to recognize cognitive impairment of WMH is not as strong as MD.

This study has some limitations. First, we originally intended to trace all the 20 fiber bundles in human brains. However, we failed to trace four of them due to technical reasons, in which case some important findings concerning tissue properties in the WMH patients could be missed. Second, due to the technical limitations, AFQ can only analyze the central portion of the WM fibers, so we cannot exclude the possibility that there exist some other portions significantly related to cognitive impairment. Moreover, this study was only cross‐sectional. If longitudinal studies can be carried out in future research, in which the clinical progress of the same subject can be traced during his lifespan, the conclusions will be more convincing. Last but not the least, the mechanisms tackling how the right IFOF and the right ILF are influenced and why they are the first two tracts to get impaired are not well illustrated in this paper. Further studies are warranted to support and substantiate findings from the present study.

## CONFLICT OF INTEREST

The authors declare that they have no conflict of interest.
