# Supplementary figures and images for: Microstructural disruption of the right inferior fronto‐occipital and inferior longitudinal fasciculus contributes to WMH‐related cognitive impairment
Source: CNS Neurosci Ther. 2020 Jan 4;26(5):576–88. doi: 10.1111/cns.13283 (PMC7163793; doi:10.1111/cns.13283)

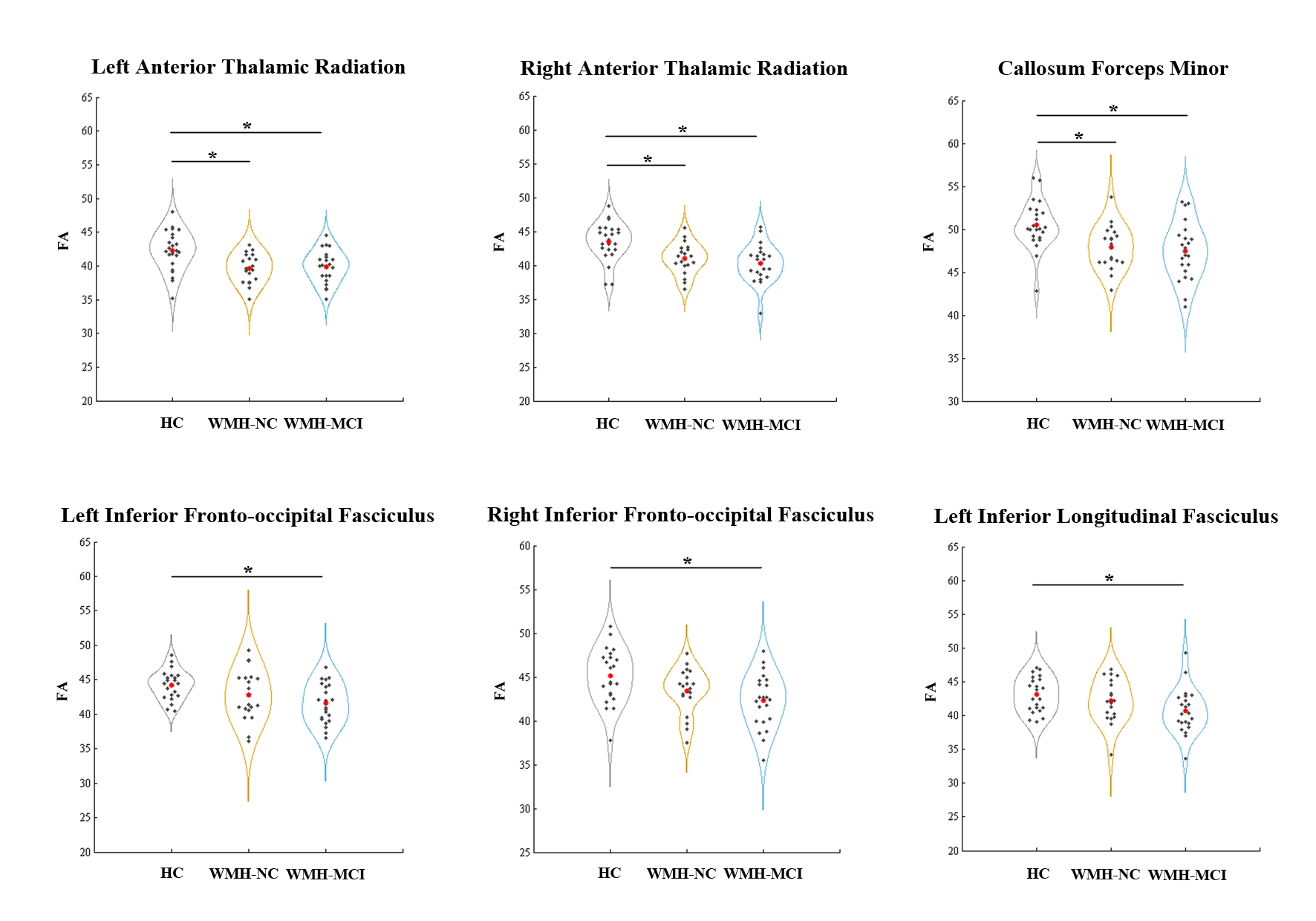

Supplement: Supplementary file 1 [file CNS-26-576-s001.tif]

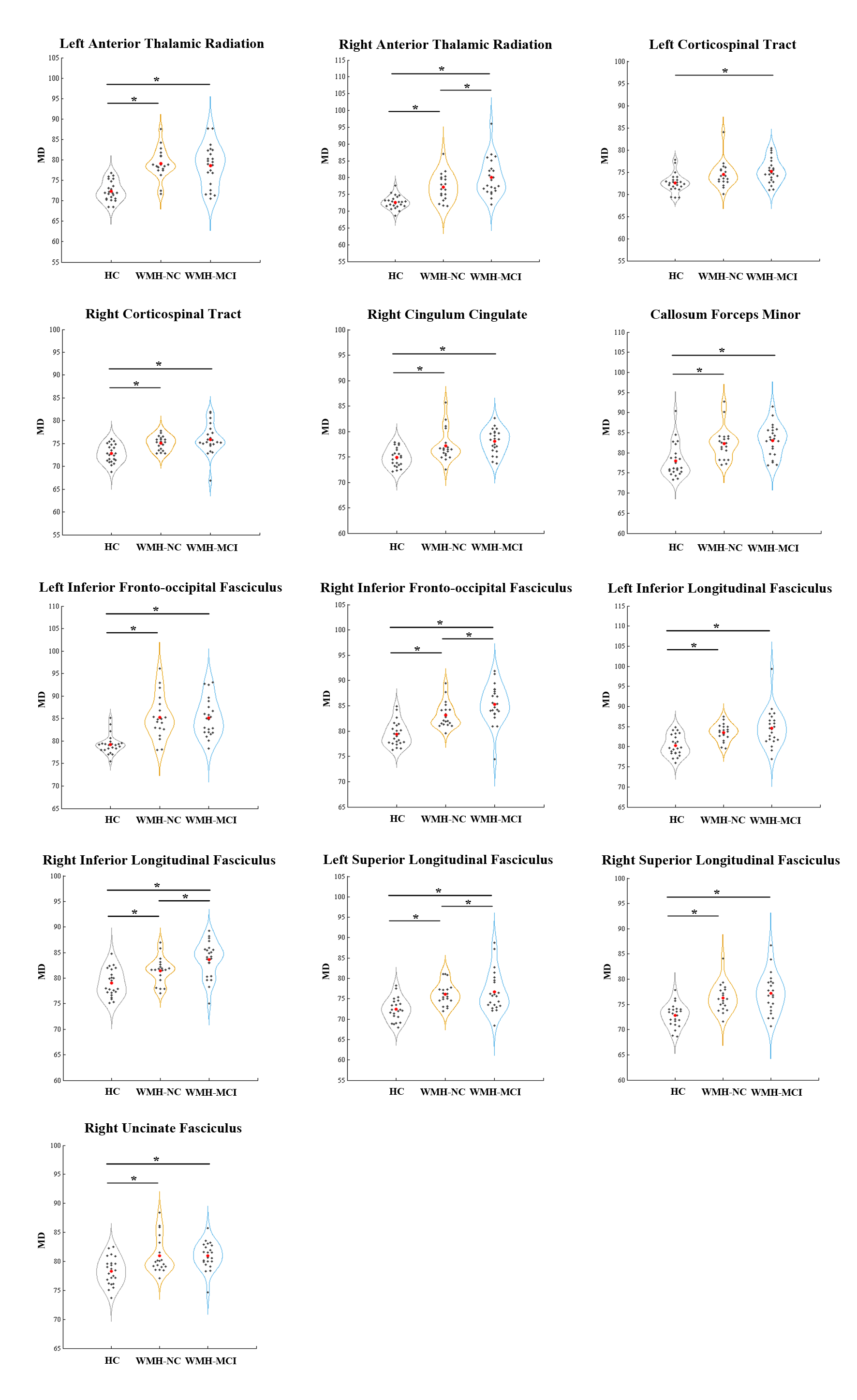

Supplement: Supplementary file 2 [file CNS-26-576-s002.tif]

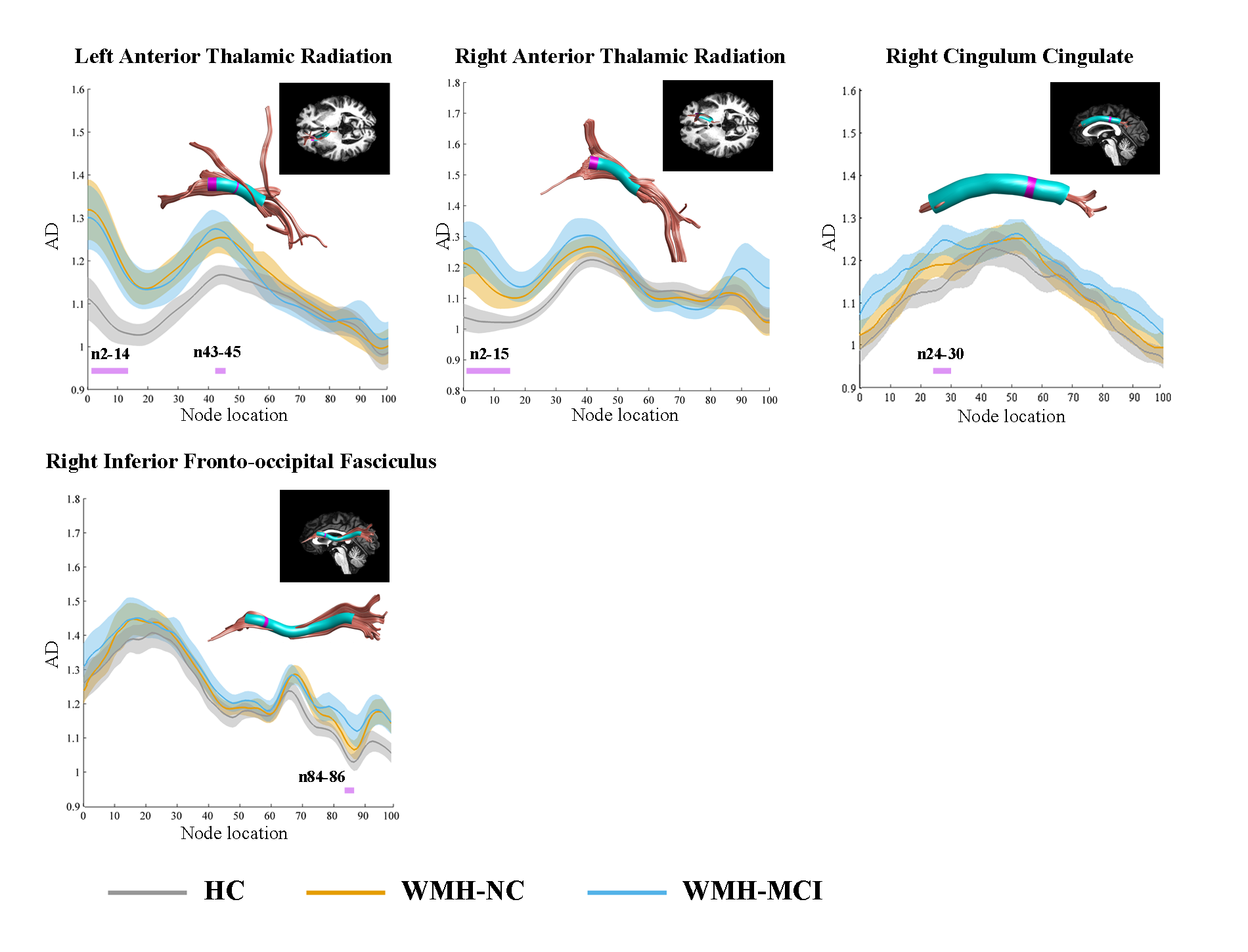

Supplement: Supplementary file 3 [file CNS-26-576-s003.tif]

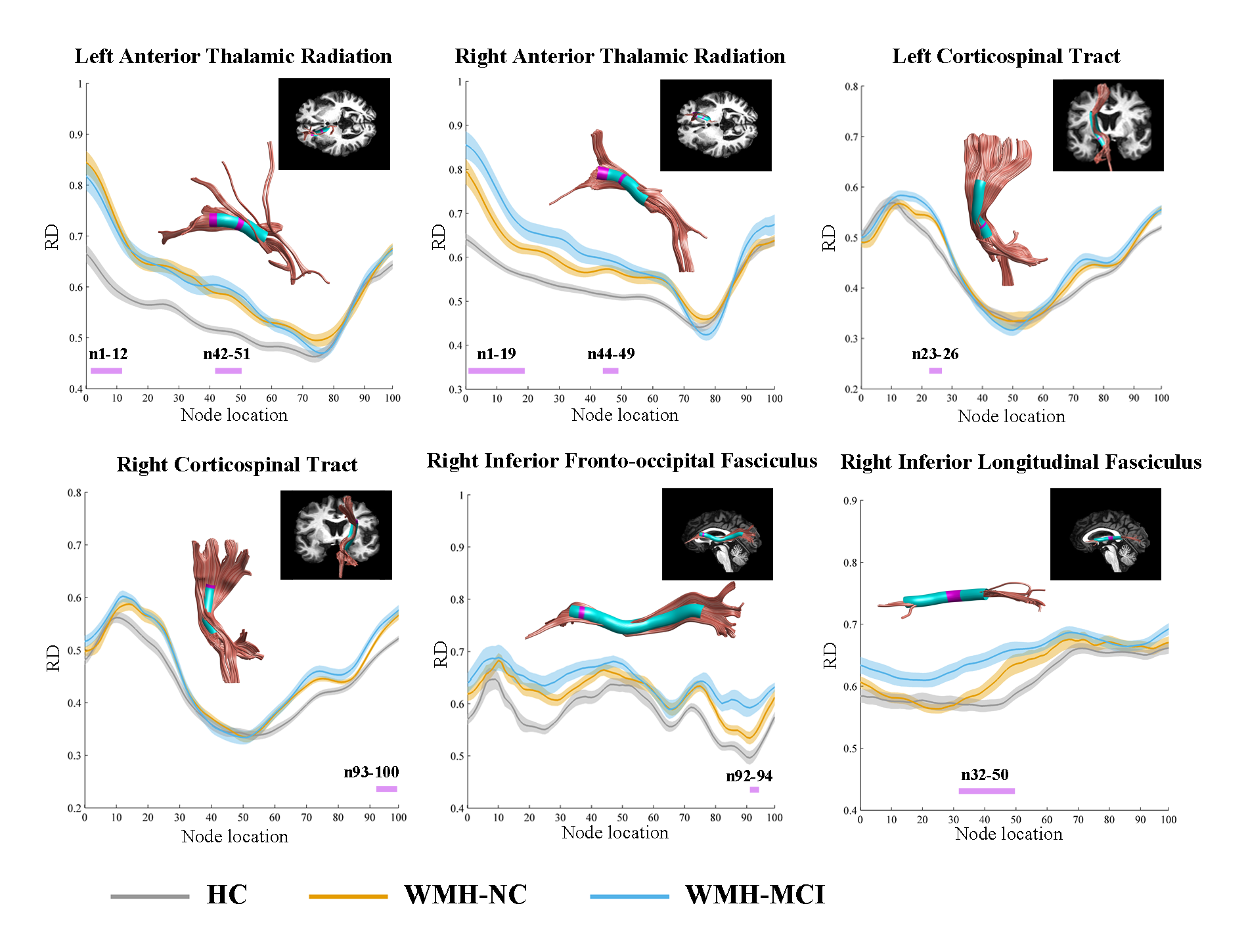

Supplement: Supplementary file 4 [file CNS-26-576-s004.tif]

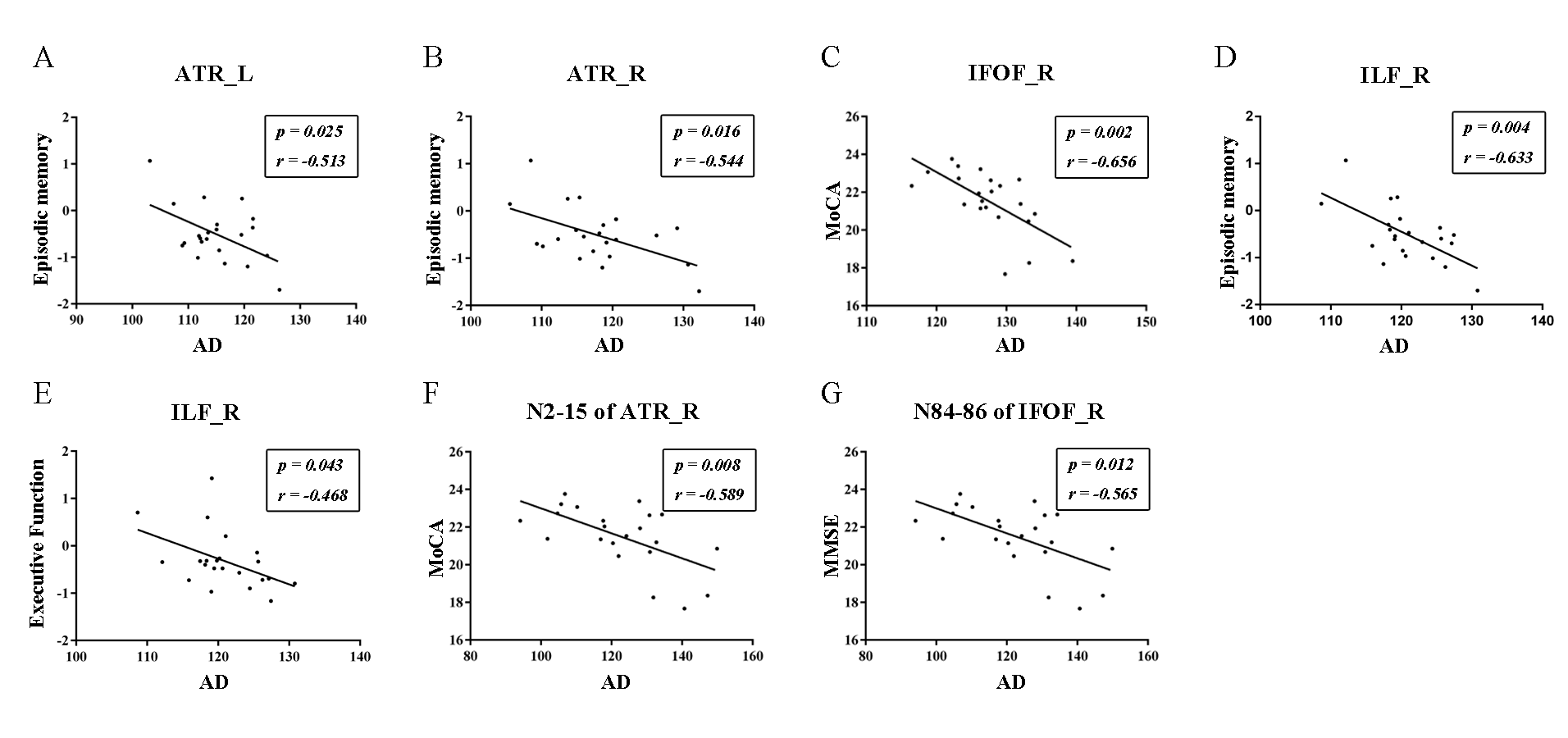

Supplement: Supplementary file 5 [file CNS-26-576-s005.tif]

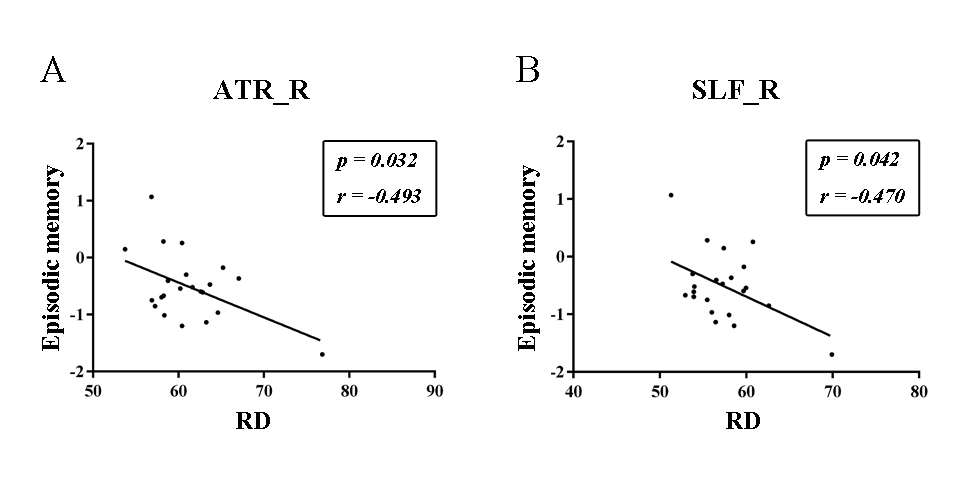

Supplement: Supplementary file 6 [file CNS-26-576-s006.tif]

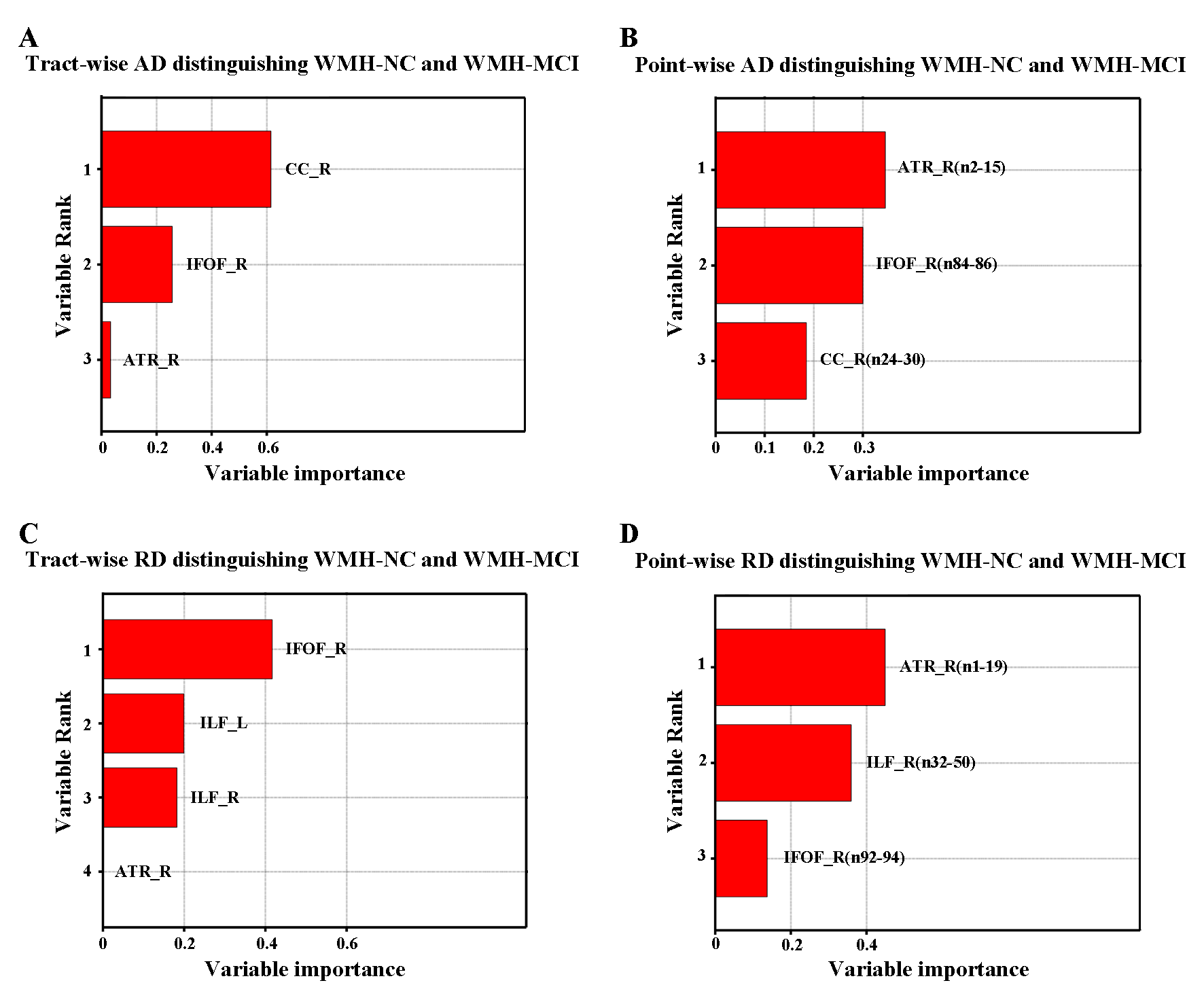

Supplement: Supplementary file 7 [file CNS-26-576-s007.tif]
